# BMP6, a potential biomarker of inflammatory fibrosis and promising protective factor for dilated cardiomyopathy

**DOI:** 10.1186/s13020-025-01062-9

**Published:** 2025-01-15

**Authors:** Feng Jiang, Jiayang Tang, Xiaoqi Wei, Hai Pan, Xinyi Fan, Peng Zhang, Shuzhen Guo

**Affiliations:** 1 School of Traditional Chinese Medicine, Beijing University of Chinese Medicine, Beijing, 102488 China; 2 Wuhan Hospital of Traditional Chinese Medicine, Wuhan, 430014 China

**Keywords:** BMP6, Dilated cardiomyopathy, Cardiac fibrosis, Heart failure

## Abstract

**Background:**

Dilated cardiomyopathy (DCM) stands as one of the most prevalent and severe causes of heart failure. Inflammation plays a pivotal role throughout the progression of DCM to heart failure, while age acts as a natural predisposing factor for all cardiovascular diseases. These two factors often interact, contributing to cardiac fibrosis, which is both a common manifestation and a pathogenic driver of adverse remodeling in DCM-induced heart failure.

**Method:**

Bulk RNA-seq, single-cell RNA-seq, Mendelian randomization analysis, animal model construction, and BMP6 knockdown were utilized to identify and validate potential specific markers and targets for intervention in DCM heart failure.

**Results:**

We found that DCM hearts exhibit pronounced inflammatory cell infiltration and fibrosis. Both bulk RNA-seq and single-cell RNA-seq analyses revealed aberrant BMP6 expression specifically in fibroblasts. The ROC curve underscores the high specificity of BMP6 in relation to DCM, while Mendelian randomization analysis further confirms BMP6 as a protective factor against DCM. Notably, BMP6 knockdown led to a decrease in SMAD6 expression and a marked elevation in COL1A1 expression levels, indicating its antifibrotic role.

**Conclusion:**

BMP6 emerges as a promising biomarker for DCM, and its functional role in exerting an antifibrotic effect underscores its potential as a therapeutic target.

**Supplementary Information:**

The online version contains supplementary material available at 10.1186/s13020-025-01062-9.

## Introduction

Heart failure (HF) is the terminal stage of various cardiovascular diseases, affecting the quality of life and life safety of approximately 60 million patients worldwide [1]. Apart from age, which is a natural risk factor [2, 3], HF is typically a consequence of multiple intertwined risk factors.

Dilated cardiomyopathy (DCM) is a common cause of HF, and its severity often necessitates heart transplantation [4]. DCM is characterized by ventricular dilation and decreased contractility of ventricular muscles [5]. The incidence of DCM lacks precise and reliable epidemiological data, and reports suggest that about 5–7 cases per 100,000 people annually are affected [6, 7]. However, this figure is likely underestimated, particularly in underdeveloped countries and regions. Arrhythmias and heart failure are the primary causes of death in DCM patients [8]. Up to 70% of DCM patients eventually succumb to heart pump failure, while 30% die suddenly from malignant arrhythmias [9]. Genetically, 40% of DCM patients exhibit abnormal gene expression, and immunologically, 66% develop cardiac-specific antibodies [9–11]. To date, over 100 aberrantly expressed genes associated with DCM, including TTN, TNN (TNNT2, TNNC1, TNNI3), and DSP, have been identified [12, 13]. Nevertheless, there remains a lack of significant breakthroughs in DCM treatment strategies, and conventional symptomatic treatments like cardiac load reduction and antiarrhythmic therapy are ineffective in improving long-term prognosis.

Currently, it is generally believed that the ultimate cause of DCM is a combination of genetic (genotype) and acquired (environmental) factors [5, 12]. In fact, the pathological mechanism of DCM encompasses multiple pathways, with inflammatory imbalance and fibrosis remodeling being key among them. The inflammatory imbalance, marked by abnormal expression of inflammatory factors and immune cell activation, is closely tied to the progression of DCM heart failure, and the elevated levels of circulating inflammatory cytokines in heart failure patients have been well-documented [14, 15]. The abnormal activation of immune cells accelerates the progression of chronic inflammatory response in DCM, leading to chronic cardiac fibrosis and and myocardial remodeling [16–18]. While anti-inflammatory therapy holds significance in cardiovascular disease treatment [19] and there are numerous general inflammatory targets such as TNF-α and IL1β, they lack tissue specificity. In terms of diagnosis, early-stage DCM patients may be found to have ventricular dilation during accidental medical examinations such as physical examinations. Despite their relatively good cardiac function at this time, there are still no targeted interventions to prevent the occurrence of heart failure. The fundamental reason for the lack of targeted treatment measures for DCM lies in the absence of reliable and effective intervention targets, as well as biomarkers for early diagnosis and prognosis evaluation. Hence, it is imperative to identify more specific biomarkers or targets of DCM to facilitate the discovery of critical and effective therapeutic pathways.

In this study, we employed a multi-genomics approach, leveraging the DCM background, to search for potential interventional target loci. We clarified the risk nature of these interventional loci through mendelian randomization analysis and validated our findings through animal model construction and gene knockdown experiments.

## Materials and methods

### Bulk RNA sequencing analysis

#### Transcriptomics analysis and weighted gene co-expression network analysis

The sequencing expression matrices of cardiac tissue from DCM patients and normal human (NH) were obtained from the GEO database (GSE29819) [20]. After normalizing the expression matrices of the two sample groups, we used the limma tool in R software to identify differentially expressed genes (DEGs) in DCM patient cardiac tissue. The following criteria were used for filtering: adjusted P-value < 0.05 and |log2FC|> 1.

We conducted weighted gene co-expression network analysis (WGCNA) on the gene expression matrices of the two sequenced groups, utilizing the disease status as a phenotype, in order to identify the most relevant gene modules for DCM. We set the key parameters for the WGCNA one-step analysis: softthreshold = 4, minModuleSize = 30, verbose = 3.

#### Assessment of immune cell infiltration abundance and differential expression of immune-related genes

The digital Cytometer-Cibersort [21], a tool that utilizes deconvolution analysis of 22 immune cell types, was used to assess the immune cell infiltration abundance in the two sample groups. By comparing the differences in immune cell infiltration abundance between the two groups, abnormal immune cell types infiltrating the cardiac tissue of DCM patients were identified. The immune-related gene set was obtained from the ImmPort database. This gene set was intersected with the DEGs to identify differentially expressed genes related to immunity (ImmDEGs) in DCM.

#### Senescence-related differentially expressed genes and significant genes and ROC sensitivity and specificity analysis

A set of aging-related gene sets [22] was matched with the DEGs to obtain differentially expressed genes related to senescence (SenDEGs) in DCM. We constructed a venn diagram among the WGCNA module closely related to DCM, ImmDEGs, and SenDEGs, where the overlapping region was considered to be the significant genes that may be closely associated with DCM. The specificity and sensitivity of the significant genes for DCM were analyzed using ROC curves.

### Single-cell RNA sequencing analysis

#### Data quality control and dimensionality reduction analysis

The dataset was obtained from the GEO database (GSE145154) [23]. We first performed data quality control on 15 samples (including 5 heart samples from normal controls and 10 heart tissue samples from patients with dilated cardiomyopathy) with the following screening criteria: nFeature_RNA > 300 & nFeature_RNA < 5000, mt_percent < 10, HB_percent < 3, nCount_RNA > 1000 & nCount_RNA < 97% quantile. After subsequently scaling and normalizing the data (method = vst, nfeatures = 3000, npcs = 50), PCA dimension reduction was performed, and the batch effect among samples was removed using the Harmony Function.

#### Annotation of cellular population

The appropriate number of dimensions was determined by constructing an elbow plot (with ndims = 50) to facilitate the distinction of cell clusters using TSNE and UMAP methods. The individual cell populations were then annotated based on the presence of different cell-specific markers in the heart.

#### Differential expression analysis of genes in cellular populations

We first observed the expression levels of potentially significant genes across various cell clusters. Subsequently, we extracted the cell clusters expressing these potentially significant genes and performed differential expression analysis between groups (DCM vs. NH), applying the same screening criteria as mentioned above (|logfc.threshold|> 1 and *P* value_adj < 0.05).

#### Differences in infiltration of macrophage subpopulations

We extracted macrophage population for deeper cellular subpopulation annotation. On this basis, we compared the differences in infiltration of these macrophage subpopulations between DCM and NH.

### Analysis of Mendelian randomization

GWAS data on exposure factors (significant genes) and outcomes (DCM) were obtained through the IEU Open GWAS database. The Mendelian randomization (MR) analysis was conducted using the TwoSampleMR package in the R software to observe the association between the significant gene and DCM. We first screened SNPs associated with significant gene (P < 5e-06), and then selected the Inverse Variance Weighted (IVW), MR Egger, Weighted Median, Simple Mode, and Weighted Mode methods to evaluate the relationship between exposure factors and outcomes, with IVW being the dominant method. We also performed heterogeneity (Cochran’s Q test) and pleiotropy of the model, and P < 0.05 was considered statistically significant. The leave-one-out method was used to assess the influence of a single SNP on the results of MR analysis.

### Construction and validation of in vivo model

#### Construction of DCM rat model

Eight 6-week-old (180 g) SPF-grade SD rats (Beijing SPF Biotechnology Co., Ltd.) were used to establish the DCM model, five rats of the same specifications served as controls. All animals were housed in the SPF animal room of Beijing University of Chinese Medicine. The specific standards of this animal room are in line with national SPF animal room standards, including a temperature range of 20 °C to 26 °C with temperature difference of less than or equal to 4 °C, a relative humidity maintained between 40 and 70%, a lighting cycle of 12 h of light alternating with 12 h of dark, and a noise level of less than 55 dB. We first constructed the DCM model by administering doxorubicin (DOX, B23031137, Aladdin, China) via intraperitoneal injection to the rats for 10 weeks. Based on published reports and our previous experimental experience, we chose an intraperitoneal injection intervention method with a dose of 2.5 mg/kg per week (with the weekly dosage divided into two equal interventions) [24]. Normal control (NC) rats were injected intraperitoneally with an equal volume of 1xPBS.

#### Cardiac function examination and heart of histopathological staining

The rats were anesthetized by intraperitoneal injection of tribromoethanol at a dose of 5 ml/kg (2.5 g of tribromoethanol dissolved in 1.25 ml of tertiary amyl alcohol and diluted to 100 ml), and after shaving the chest hairs, cardiac function was carried out by echocardiography (Vevo2100, Visual Sonics, Canada). Subsequently, the rats were euthanized, and part of the heart was fixed in paraformaldehyde for paraffin sectioning. ELISA kit (AE2940A, Creargent, Tianjin, China) was used to detect the changes of NT-proBNP in serum.

For the Hematoxylin and Eosin (HE) staining, we first place the paraffin sections in an oven at 65 °C for 30 min for baking, followed by dewaxing with xylene (repeated three times, 10 min each), 100% ethanol (repeated twice, 5 min each), 90% ethanol (5 min), and 80% ethanol (5 min) in sequence. Rinse with distilled water for 3 min. Subsequently, immerse the sections in hematoxylin staining solution for 3 ~ 5 min, rinse three times with distilled water for 3 min, and then place them in 1% hydrochloric acid alcohol for approximately 15 ~ 30 s, rinse three times with distilled water for 3 min. Remove the sections, and drop eosin staining solution for 3 ~ 5 min. Subsequently, dehydrate the sections by placing them in 75% ethanol, 90% ethanol, 95% ethanol, and 100% ethanol for 2 min each. Finally, place the sections in xylene three times, 2 min each, and seal the slides with neutral resin.

For the Masson staining, the paraffin sections were placed in an oven at 65 °C for 2 h to bake. The dewaxing procedure for these sections is similar to that of HE. After dewaxing, immerse the sections in 2.5% potassium dichromate mordant overnight. The next day, place the sections in hematoxylin dye solution for 5 min, rinse with distilled water for 5 times, and then place them in 1% hydrochloric acid alcohol for about 15 s before removing the sections. Then place the sections in the ponceau acid fuchsin staining solution for about 10 min, rinse with distilled water, place them in 1% phosphomolybdic acid solution for about 1 min, then immerse the sections in aniline blue solution for staining for about 30 s, and rinse three times with 1% glacial acetic acid aqueous solution. The dehydration and sealing steps are similar to HE.

#### Validation of transcriptomics sequencing analysis and immunofluorescence analysis and western blot

0.5 g of each heart tissue sample was excised for Bulk RNA-seq analysis (Illumina platform provided by Personal Gene Technology CO., LTD, Shanghai, China). Differential expression analysis was conducted utilizing the DESeq2 R package under the same preset conditions as mentioned previously. For immune cells, the TSA triple immunofluorescence staining kit (RS0036, ImmunoWay, USA) was used to detect specific biomarkers. DAPI (P0131, Beyotime Biotech, China) was used to stain the cell nucleus, F4/80 (28463-1-AP, Proteintech, USA) to stain macrophages, and CD206 (18704-1-AP, Proteintech, USA) to stain M2 macrophages. Additionally, the expression levels of proteins, including BMP6 (M06924-1, Boster, USA), SMAD3 (25494-1-AP, ProteinTech, USA), SMAD6 (sc-25321, Santa Cruz, USA), IL-1β (16806-1-AP, ProteinTech, USA), IL-18 (60070-1-Ig, ProteinTech, USA), IL-6 (21865-1-AP, ProteinTech, USA), TNF-α (17590-1-AP, ProteinTech, USA), and COL1A1 (sc-293182, Santa Cruz, USA), were detected using western blotting. The results from repeated detections in three independent samples were statistically analyzed (n = 3).

### Knockdown of BMP6 and western blot validation and prediction of potential herbal medicines

#### Extraction of primary fibroblasts

Ten 2–3-day-old SD rats were selected, sterilized, and sacrificed to quickly remove their hearts, which were then rinsed twice in D-Hanks solution. The hearts were subsequently transferred to a 6-cm petri dish placed on an ice box, cut into pieces using scissors, and treated with an appropriate amount of trypsin (adjusted to a concentration of 0.05%) for overnight digestion in a 4 °C refrigerator. Following digestion, complete culture medium was added to terminate the enzymatic reaction. Subsequently, type II collagenase (adjusted to a concentration of 0.2%) was added for an additional 30 min of digestion, followed by filtration. The filtrate was centrifuged, and the precipitate was resuspended in complete culture medium before being seeded into a 6-well plate for 2 h to allow for differential adherence. The adherent cells were primary fibroblasts.

#### Knockdown of BMP6 via siRNA transfection and detection of western blot

When the cell growth fusion reaches 70%, siRNA (Suzhou GenePharma Co., Ltd., Suzhou, China) is transfected to knockdown BMP6 expression in primary fibroblasts. Following the operation manual, after 6 h of siRNA transfection (Sense 5′–3′: GCAGCAGCAACAAUCGCAATT; Antisense 5′–3′: UUGCGAUUGUUGCUGCUGCTT), the complete medium is replaced for further cultivation. At 24 h post-transfection, proteins are extracted for western blot detection.

#### Reverse prediction of herbal medicines regulating BMP6

The HERB database was used to perform reverse prediction of herbal medicines capable of regulating BMP6. The herbal medicines predicted to have an FDR value < 0.05 were considered as the potential target medicines.

### Statistical analysis

Data are expressed as mean ± standard deviation. Unpaired t-test was used for differences between groups and paired t-test for differences within groups. Statistical analyses and histogram construction were performed by GraphPad Prism 8.0 and *P* < 0.05 was considered statistically significant.

## Results

### Bulk RNA sequencing analysis

#### Transcriptomics analysis and WGCNA

**Fig. 1:**
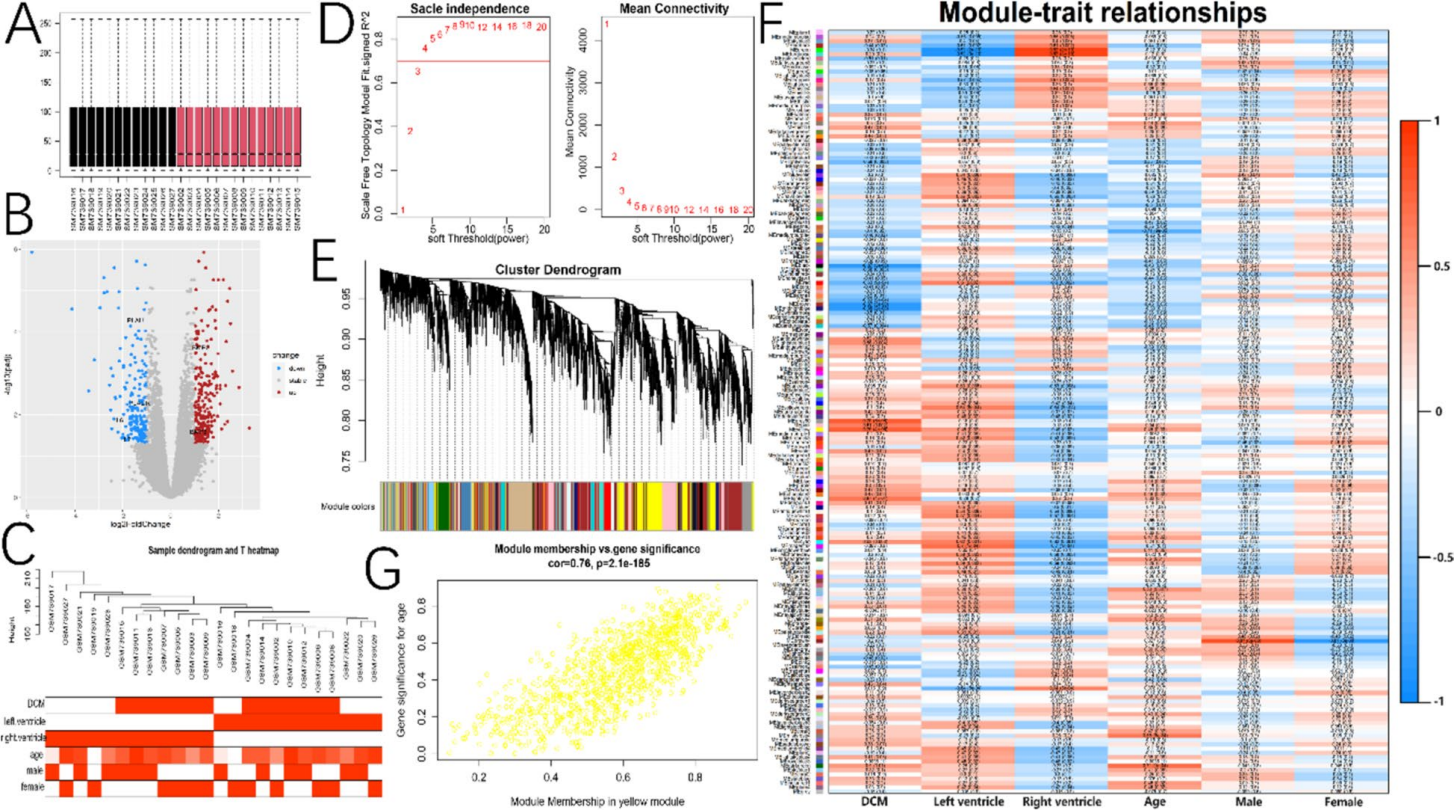
Differential expression analysis and WGCNA. **A** Normalization between samples. **B** Differential expression analysis of bulk RNA-seq sequencing results between DCM and NH. **C** Sample discrete dendrogram and heat map of disease trait distribution. **D** Scale independence and mean connectivity change curves and soft-threshold cutoff. **E** Gene module clustering dendrogram. **F** Correlation between DCM phenotypic characteristics and gene modules. **G** Correlation between yellow module membership and gene significance for DCM

Differential expression analysis revealed 193 downregulated DEGs (e.g., IL6, IL7, PLAU) and 244 upregulated DEGs (e.g., BMP6, BMP2, CXCR6*)* in the cardiac tissue of DCM patients (Fig. 1A and B). In the result of WGCNA, after performing clustering analysis on the two sample groups (Fig. 1C), a soft threshold was determined (power = 4) (Fig. 1D), and gene modules associated with each characteristic were identified based on this soft threshold (Fig. 1E). Among these modules, the MEyellow module showed the strongest positive correlation with DCM (Fig. 1F), and there was a significant correlation between module membership and gene significance within this module (Fig. 1F and G).

#### Immune cell infiltration abundance and ImmDEGS

**Fig. 2:**
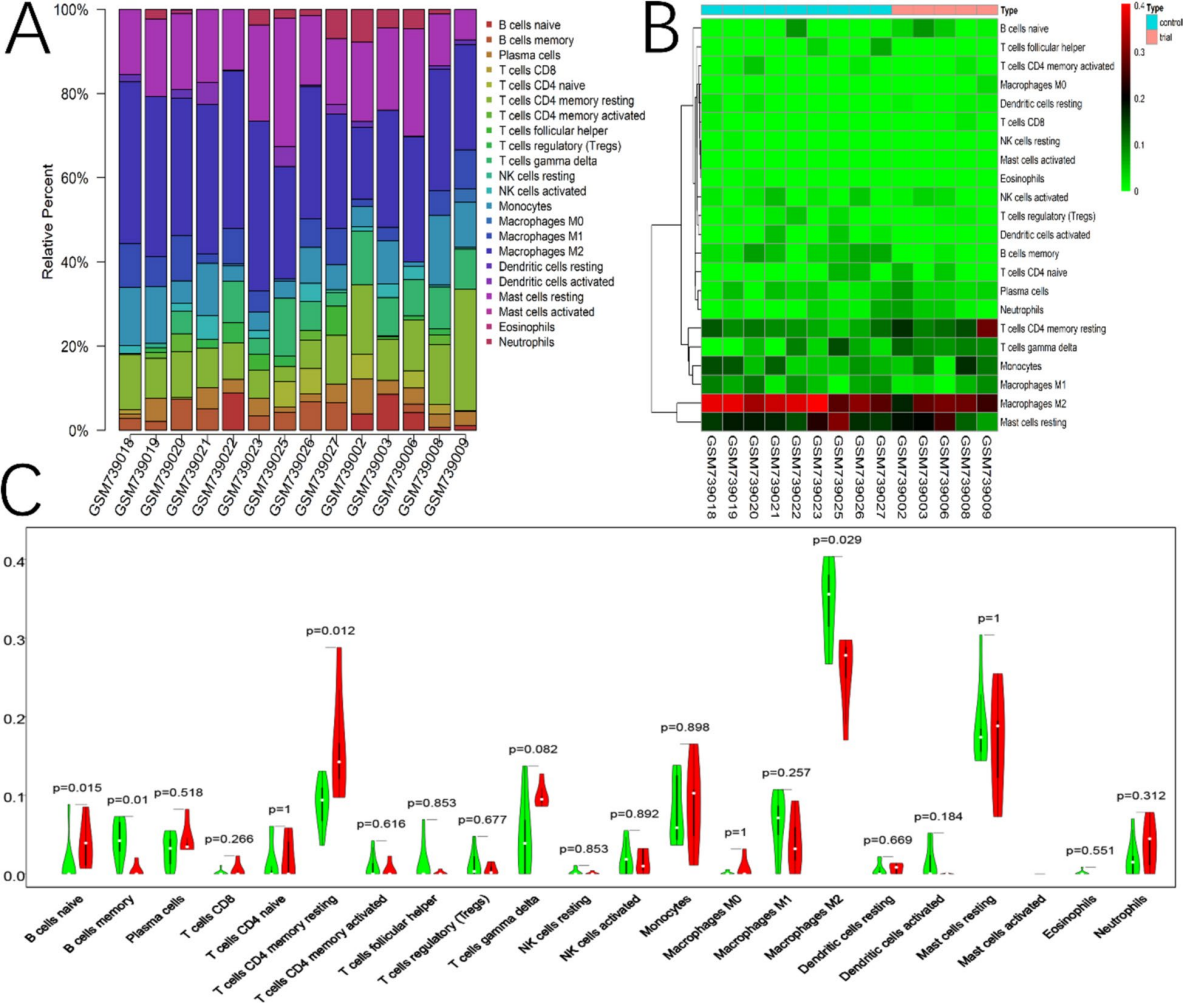
Assessment of cardiac immune response in DCM. **A** Differences in the abundance percentage of 22 immune cells across sample species. **B** Differences in the infiltration of different immune cells in each sample. **C** Differences in immune cell infiltration between DCM and NC

**Fig. 3:**
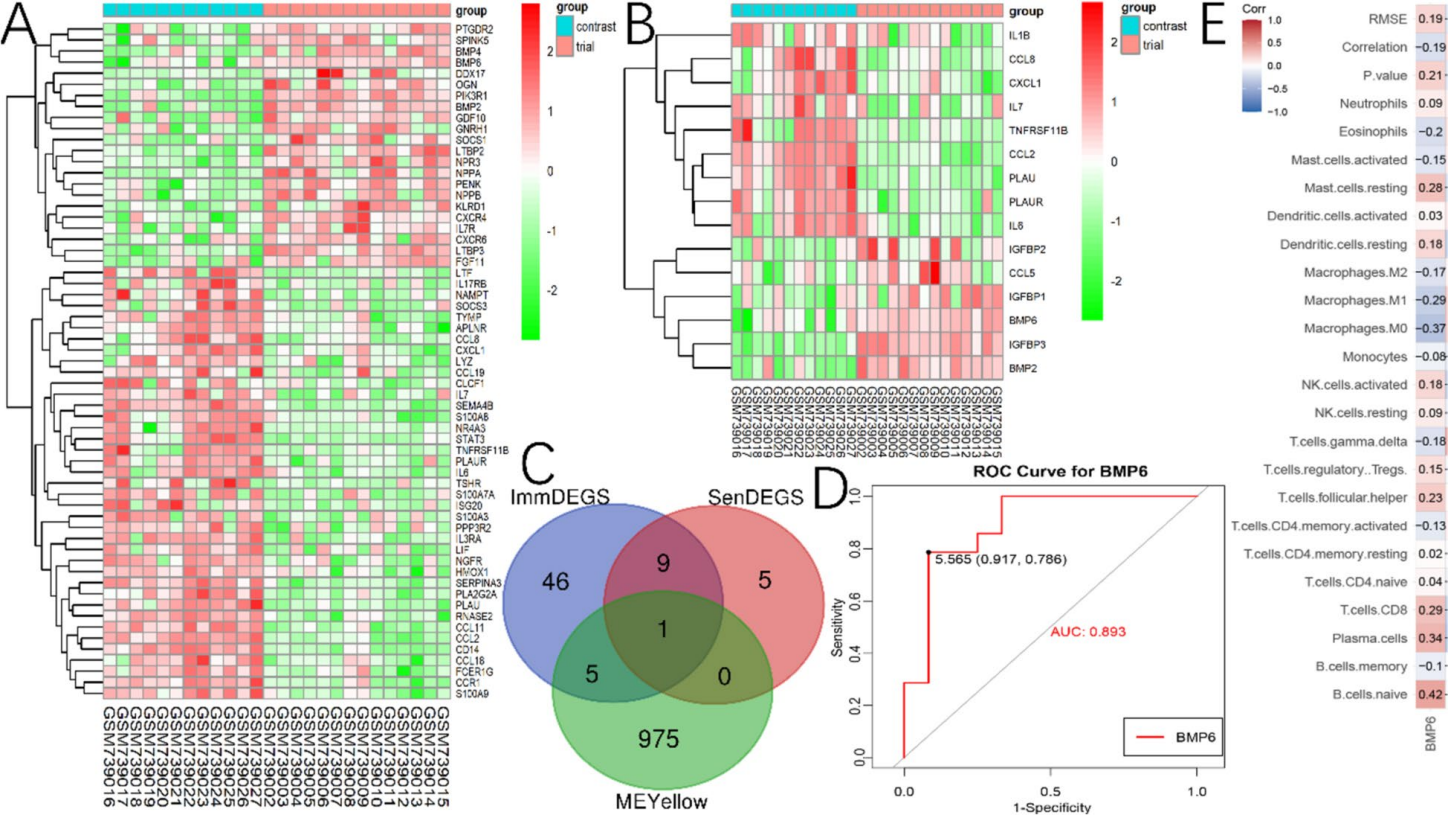
Significant gene and assessment of biomarker sensitivity. **A** Heatmap of differential expression of immune-related genes between DCM and NC. **B** Heatmap of differential expression of senescene-related genes between DCM and NC. **C** Intersection of ImmDEGS, SenDEGS and gene sets in MEyellow. **D** Sensitivity and specificity analysis of ROC curves between BMP6 and DCM. **E** Correlation between BMP6 and 22 types of immune cells

We found differences in immune cell infiltration abundance between DCM and NH in cardiac tissue (Fig. 2A–C), which suggests the presence of an abnormal inflammatory response in DCM hearts. At the level of immune-related genes, we discovered that 61 ImmDEGs were aberrantly expressed in the cardiac tissue of DCM patients. Among these, 22 ImmDEGs (e.g., BMP6, BMP2, BMP4, CXCR4) were upregulated, whereas 39 ImmDEGs (e.g., IL7, IL6, CCL2, PLAUR) were downregulated (Fig. 3A).

#### SenDEGS and significant genes and ROC sensitivity and specificity analysis

We also found that 15 Sen-DEGs were aberrantly expressed in the cardiac tissue of DCM patients, with 6 Sen-DEGs (e.g., BMP6, IGFBP1, IGFBP2, IGFBP3) being significantly upregulated, and 9 Sen-DEGs (e.g., IL6, IL7, PLAUR) being significantly downregulated (Fig. 3B). We obtained an overlapping gene, BMP6, by intersecting the lists of ImmDEGs, SenDEGs, and the genes within the MEyellow module (Fig. 3C). The ROC curves demonstrated that BMP6 (AUC = 0.893) exhibits good specificity and sensitivity for DCM (Fig. 3D). Furthermore, we observed a correlation between BMP6 and immune cells, specifically a negative correlation with M2 macrophages (Fig. 3E).

### Single-cell RNA sequencing analysis

#### Data quality control and dimensionality reduction analysis

After processing the data with quality control measures and applying PCA for dimensionality reduction, the batch effect was mitigated by utilizing the Harmony Function (Figure S1).

#### Annotation of cellular populations

**Fig. 4:**
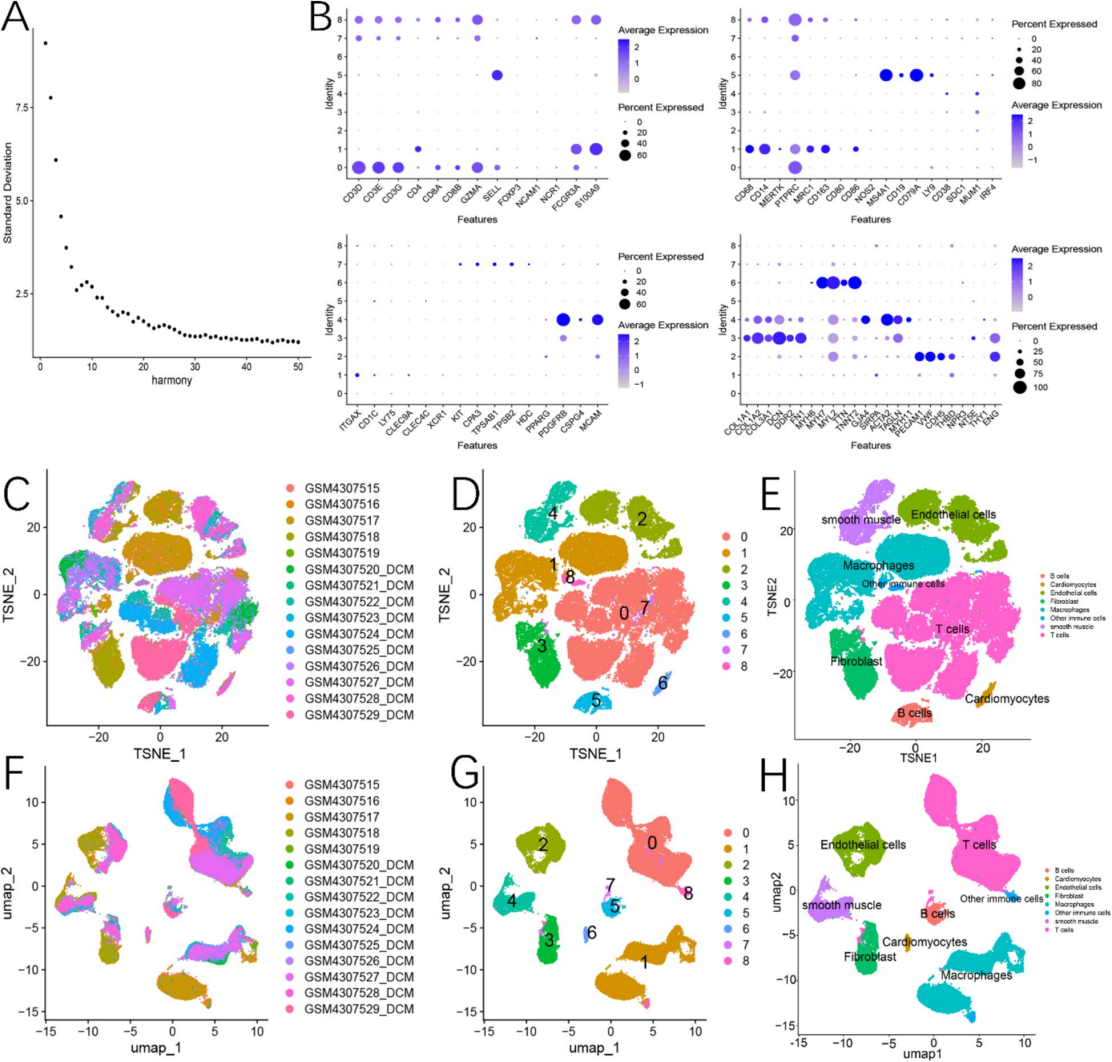
Cell clustering and annotation. **A** Elbow diagram obtained using the harmony method and we selected 0–22 as the number of dimensions. **B** Specific markers and expression levels in different cardiac cell populations. **C**–**E** Subdividing and annotating cardiac cell populations by TSNE method. **F**–**H** Subdividing and annotating cardiac cell populations by Umap method

Based on the elbow diagram (dimensions 0–22) (Fig. 4A), nine cell clusters were subsequently identified and annotated into eight distinct cell types using specific marker genes (Fig. 4B). These cell types included T cells, B cells, macrophages, endothelial cells, fibroblasts, cardiomyocytes, smooth muscle cells, and a category labeled as other immune cells (Fig. 4C–H).

#### Differential expression analysis of genes in cellular populations between DCM and NH

**Fig. 5:**
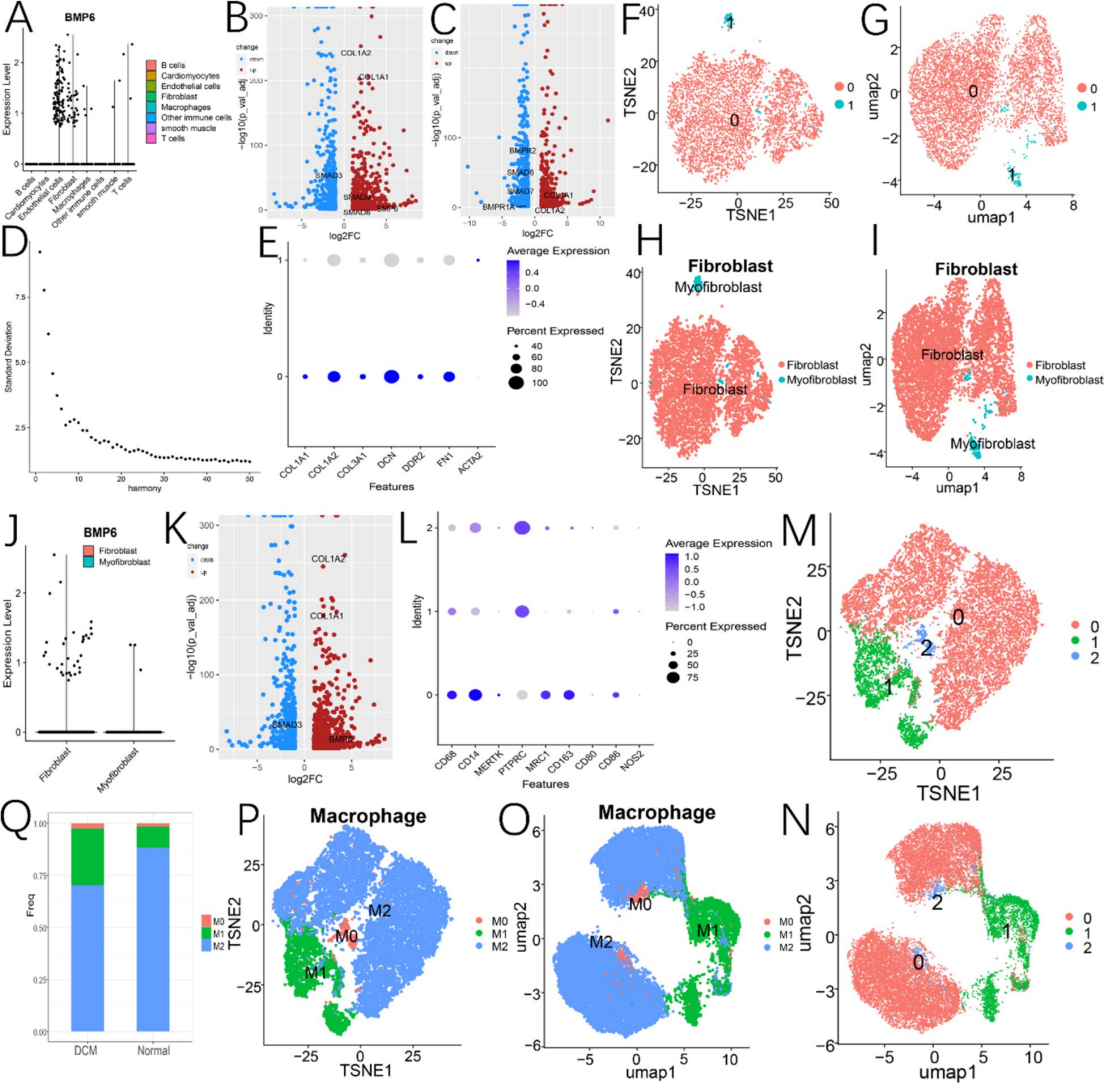
BMP6 expression in different cellular subpopulations. **A** Differential expression of BMP6 in various cell types of the heart. **B** Differential gene expression in fibroblasts between DCM and NH. **C** Differential gene expression in endothelial cells between DCM and NH. **D** Elbow diagram obtained using the harmony method and we selected 0–15 as the number of dimensions. **E** Specific markers and expression levels in fibroblasts and myofibroblasts. **F**–**I** subdividing and annotating fibroblast subpopulations by TSNE and Umap methods. **J** BMP6 expression between conventional fibroblasts and myofibroblasts. **K** differential expression of BMP6 in fibroblasts between DCM and NH. **L** Specific markers and expression levels of macrophage subpopulations. **M**–**P** Subdividing and annotating macrophage subpopulations by TSNE and Umap methods. **Q** Differences in the percentage of macrophage subpopulations abundance between DCM and NH

Our analysis revealed that BMP6 is predominantly expressed in endothelial cells and fibroblasts, with minimal expression in other cardiac cell types (Fig. 5A). When comparing the differentially expressed genes between DCM and NH specifically in fibroblast and endothelial cell clusters, we found that BMP6 was significantly overexpressed only in fibroblasts from DCM compared to NH, while no significant difference was observed in endothelial cells between the two groups. Additionally, we noted that the expression of COL1A1 and COL1A2 in both fibroblast and endothelial cell clusters of DCM was significantly higher than in NH (Fig. 5B and C). Consistent with the expression pattern of BMP6, SMAD6 and SMAD9 were significantly overexpressed in fibroblasts of DCM patients compared to NH. Interestingly, however, SMAD6 was significantly underexpressed in endothelial cells of DCM patients compared to NH. Upon further subdivision of fibroblasts into ordinary fibroblasts and myofibroblasts (Fig. 5D–I), we found that BMP6 was highly expressed only in conventional fibroblasts of DCM patients, with no significant difference observed in myofibroblasts between the two groups (Fig. 5J and K).

#### Differences in infiltration of macrophage subpopulations between DCM and NH

After isolating the macrophage population, it was further subdivided into three distinct subpopulations, and a thorough annotation of these subpopulations was performed using specific markers (Fig. 5L). As a result, three subpopulations were identified: M0 macrophages, M1 macrophages, and M2 macrophages (Fig. 5M–P). We then compared the infiltration patterns of these macrophage subpopulations between DCM and NH. Our findings revealed that the proportion of M2 macrophages was significantly lower in DCM compared to NH (Fig. 5Q).

### Assessment of Mendelian randomization

**Fig. 6:**
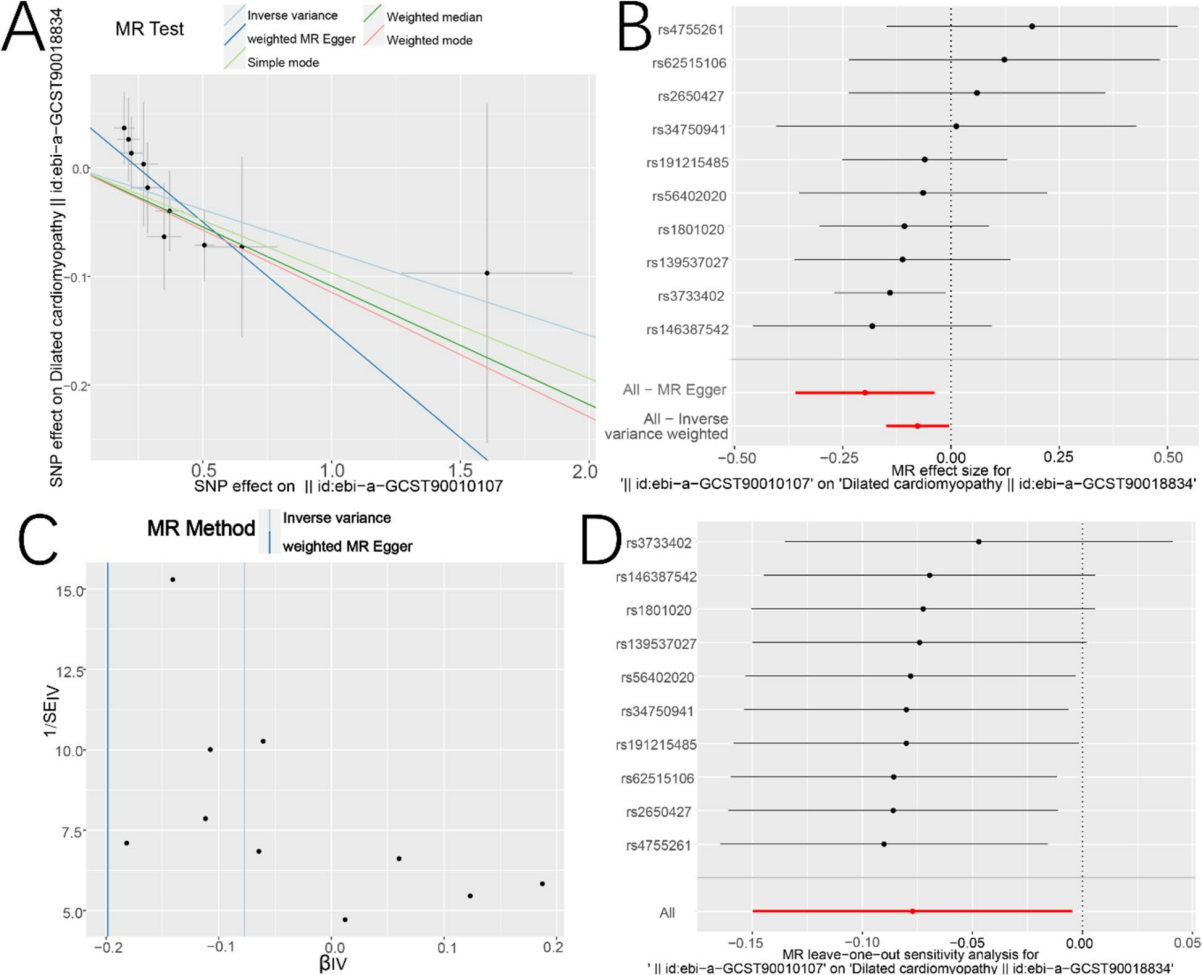
Mendelian randomization analysis between BMP6 and DCM. **A** Scatter trend plot of effective SNPs. **B** The black line represents the nature of each SNP, and the red lines represent the combined nature of the SNPs analyzed by the IVW and MR Egger methods, respectively. **C** SNPs distribution map. **D** Leave-one-out analysis. The red line shows the results of the combined analysis

We conducted a Mendelian Randomization (MR) assessment between the exposure factor (id: ebi-a-GCST90010107) and the outcome (id: ebi-a-GCST90018834). The IVW estimation method (beta = − 0.138, OR = 0.872, *P* value = 0.007) and the weighted median method (beta = − 0.136, OR = 0.873, *P* value = 0.013) suggested that BMP6 is a protective factor for DCM (Table 1 and Fig. 6A–C). The leave-one-out analysis results showed that all SNP points were on the left side of the zero-cut-off line, and removing each SNP individually did not have a substantial impact on the results (Fig. 6D). This suggests that the relationship between BMP6 and DCM is sensitive.

**Table 1:** Mendelian randomization method of estimation

Method	nSNP	Beta	SE	Pval	OR	OR_lci95	OR_uci95
Inverse variance weighted	10	− 0.138	0.051	0.030	0.941	0.890	0.994
MR Egger	10	− 0.145	0.310	0.729	0.943	0.690	1.289
Weighted median	10	− 0.136	0.055	0.082	0.937	0.871	1.008
Simple mode	10	− 0.134	0.069	0.169	0.920	0.828	1.021
Weighted mode	10	− 0.136	0.062	0.180	0.927	0.840	1.022

### Construction and validation of in vivo model

**Fig. 7:**
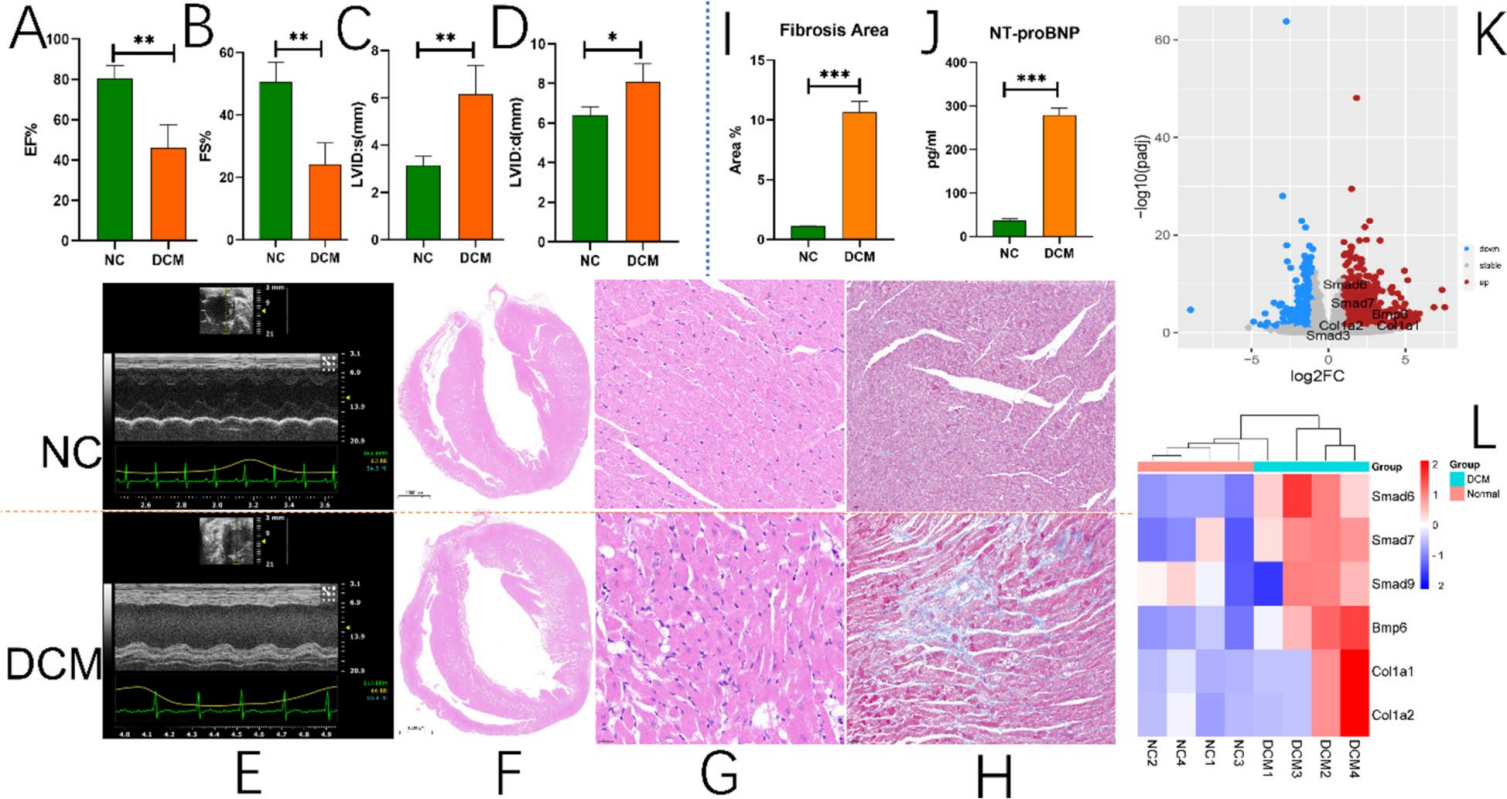
Assessment of cardiac fibrosis and RNA-Seq analysis. **A** Comparison of cardiac ejection fraction between DCM and NC rats. **B** Comparison of left ventricular fractional shortening between DCM and NC rats. **C** and **D** Comparison of left ventricular internal diameters in systole and diastole between DCM and NC rats. **E** Echocardiography testing of cardiac function. **F** Morphology of longitudinal sections of the heart under HE staining. **G** HE staining of the lateral inner edge of the left ventricular wall of the heart at 50 × magnification. **H** Masson staining of the area located at the lateral inner edge of the left ventricular wall of the heart, viewed at 20 × magnification. **I** Comparison of fibrosis area by Masson staining (n = 3). **J** ELISA detection of changes in NT-proBNP in serum (n = 4). **K** and **L** Differential expression analysis of transcriptomics results between DCM and NC rats

**Fig. 8:**
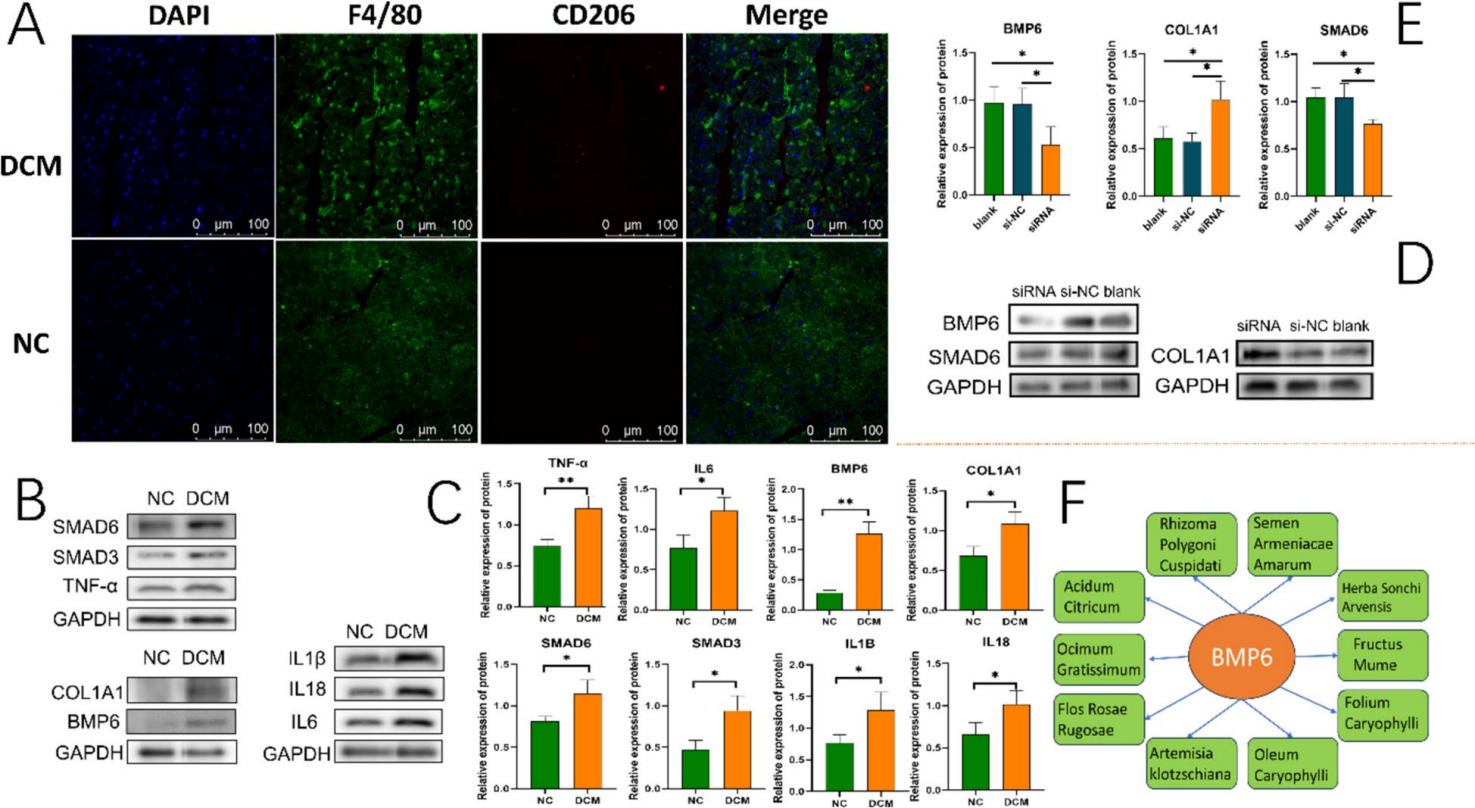
Immunofluorescence staining and cardiac tissue protein assay and BMP6 knockdown. **A** Immunofluorescence staining of markers specific to macrophages and type 2 macrophages. **B** and **C** Western blot was used to detect the changes in protein levels in the cardiac tissues between DCM and NC (n = 3). **D** and **E** The knockdown of BMP6 and the expression levels of SMAD6 and COL1A1 (n = 3). The siRNA group represents the knockdown group, si-NC represents the empty vector group, and blank represents the control group. The significance of *P in the above graph: *P < 0.05; **P < 0.01. **F** Potential interventional herbs based on BMP6 inverse prediction

The results of echocardiography examination revealed that compared to the NC group, the rats in the DCM group exhibited significantly decreased left ventricular ejection fraction (EF) and left ventricular fractional shortening (FS), as well as significantly increased left ventricular internal diameters during both diastole and systole (Fig. 7A–E). In addition, the histopathological staining results showed that the rat hearts in the DCM group were significantly dilated, accompanied by obvious inflammatory cell infiltration and fibrosis (Fig. 7F–I). We also found that the serum NT-proBNP level in DCM rats was significantly higher than that in the NC group (Fig. 7J). Transcriptomics analysis revealed that BMP6, SMAD6, SMAD7, COL1A1, and COL1A2 were significantly highly expressed in the DCM compared to the NC (Fig. 7K and L). The immunofluorescence results showed that the infiltration level of macrophages and M2 macrophages in the DCM group was significantly higher than that in the NC group, but the overall proportion of M2 macrophages in total macrophages was significantly lower than that in the NC group (Fig. 8A). In terms of protein expression in cardiac tissue, we found that the expression levels of BMP6, SMAD3, SMAD6, IL1β, IL18, IL6, TNF-α, and COL1A1 in the DCM group were significantly higher than those in the NC group (n = 3) (Fig. 8B and C).

### Knockdown of BMP6 and validation of western blot and prediction of potential herbal medicines

After knockdown of BMP6, the expression of SMAD6 was decreased, whereas the expression of COL1A1 was significantly increased (n = 3) (Fig. 8D and E). Based on BMP6, we predicted 35 potential herbal medicines that regulate BMP6 for the treatment of DCM through the HERB database, such as Rhizoma Polygoni cuspidati, Acidum Citricum, and Herba Sonchi Arvensis. According to the FDR values, we presented the top 10 potential herbal medicines (Fig. 8F).

## Discussion

DCM is one of the leading causes of HF and the main HF phenotype leading to cardiac transplantation [25, 26]. However, there remains a dearth of targeted treatment measures specifically for DCM. To date, even when patients in the early stages of DCM discover ventricular dilation only during routine medical examinations such as physical check-ups, and despite their relatively preserved cardiac function at this point, there are still no reliable and targeted interventions to halt the progression of DCM towards HF. Consequently, the identification of more specific biomarkers and therapeutic targets holds the key to effective DCM treatment.

Age is an inevitable and natural risk factor for all cardiovascular diseases, including HF [27, 28]. Significant differences in matrix protein secretion profiles have been observed between cardiac fibroblasts from older and younger animals, with aging found to exacerbate cardiac fibrosis [29]. Inflammatory responses are pervasive throughout the HF process, playing a pivotal role in regulating adverse cardiac structural remodeling and myocardial tissue fibrosis [30, 31]. Notably, fibrosis is a hallmark of most chronic inflammatory responses [32, 33], and diseases associated with aging, such as cardiovascular disease and diabetes, are frequently accompanied by tissue inflammatory fibrosis [34], which often culminates in organ failure. The excessive presence of immune cells, particularly macrophages, mast cells, and activated lymphocytes, fuels the inflammatory fibrotic process by secreting inflammatory factors like TNF-α, IL-1β, and IL-6. These inflammatory factors not only directly activate myofibroblasts to induce tissue fibrosis but also stimulate macrophages to transform into myofibroblasts, further promoting fibrosis [15–18]. Clearly, inflammatory factors serve as the bridge between these processes, and the sustained high levels of inflammatory factors in heart failure patients provide a molecular basis for cardiac fibrosis development [14]. Our histopathological analysis of hearts from DCM rats revealed a substantial infiltration of inflammatory cells. Consistent with previous reports, macrophages, lymphocytes, and mast cells are the primary immune cells implicated in cardiac fibrosis, with macrophages potentially playing a more prominent role [35]. Our analysis of immune cell infiltration using bulk RNA-seq and single-cell RNA-seq revealed that the inflammatory response in DCM hearts is abnormal, characterized by a reduced proportion of anti-inflammatory M2 macrophages compared to normal hearts (Figs. 2C and 5Q), which was confirmed in our immunofluorescence staining experiments (Fig. 8A). The crosstalk between inflammation, aging, and tissue fibrosis is a vicious cycle that poses significant challenges in treatment and prevention.

In this study, we identified a potentially significant gene, BMP6, which exhibited robust diagnostic performance for DCM through ROC curve analysis (AUC = 0.893, specificity = 0.917, sensitivity = 0.786). To gain further insights, we employed high-resolution single-cell RNA-seq and annotated the cell populations, revealing that BMP6 was exclusively expressed in endothelial cells and fibroblasts (Fig. 5A). Notably, BMP6 was aberrantly overexpressed solely in fibroblasts of DCM hearts compared to normal hearts (NH), hinting at a potential link between BMP6 and fibrosis. Given that cardiac fibrosis involves the differentiation of conventional fibroblasts into myofibroblasts, which secrete fibrotic factors like collagen, we further categorized fibroblasts into ordinary and myofibroblast subpopulations (Fig. 5E–I). Our analysis showed that BMP6 was abnormally upregulated only in conventional fibroblasts of DCM hearts (Fig. 5J and K), suggesting a potential role in regulating their differentiation. To validate our findings, we constructed a DCM rat model and observed significant cardiac fibrosis accompanied by elevated BMP6 and COL1A1 expression. BMP6, a member of the TGFβ superfamily, has been reported to exacerbate HF in cardiac infarction models upon knockdown [36]. Our MR analysis, akin to this finding, revealed that BMP6 acts as a protective factor against DCM-related heart failure (Table 1 and Fig. 6A–D).

Considering the family characteristics of BMP6, we believe that BMP6 may be related to fibrosis. Interestingly, our MR analysis and prior studies suggest that BMP6 may function analogously to BNP in heart failure, serving as an indicator of fibrosis severity (BNP is a biomarker for the degree of heart failure) while exerting an antifibrotic effect (BNP ameliorates heart failure through diuresis). To validate this, we transfected rat cardiac primary fibroblasts with BMP6 knockdown and observed a marked upregulation of COL1A1, consistent with our hypothesis. Remarkably, single-cell RNA-seq data have revealed that SMAD6, an inhibitory SMAD, is highly expressed in DCM fibroblasts compared to NC, consistent with the expression trend of BMP6. This suggests that BMP6 may counteract fibrosis by upregulating SMAD6. BMP6, as a member of the TGFβ superfamily, can also regulate smad subtypes. Within the SMAD family, SMAD can be categorized into three subfamilies, i.e., receptor-regulated Smad (R-SMAD), Common-mediator Smad (Co-SMAD), and inhibitory Smad (I-SMAD) [37–39].The R-Smad consists of SMAD1, SMAD2, SMAD3, SMAD5, and SMAD8, and the Co-SMAD is SMAD4, I -SMAD includes SMAD6 and SMAD7. Functionally, SMAD2/SMAD3 promotes fibrosis [39, 40]. SMAD6/SMAD7 exerts an anti-fibrotic effect primarily through competitive antagonism of SMAD2/SMAD3 and others [41, 42]. Conventionally, SMAD1/SMAD5/SMAD8 mainly binds with the BMP subfamily to regulate the growth and development of tissues and organs [43, 44]. However, TGFβ and BMP originate from the same large family, so these regulatory relationships are not static [40, 43]. COL1A1 and COL1A2 are the predominant direct indicators of tissue fibrosis, and high expression of SMAD6/SMAD7 typically implies the initiation of negative feedback regulation of antifibrotic mechanisms [32, 45]. Thus, we also detected changes in SMAD6 expression after BMP6 knockdown and found that SMAD6 expression levels were significantly reduced. Given that TGFβ-SMAD3 is a typical pro-fibrotic regulatory pathway, and considering BMP6's familial background, it is not difficult to speculate that BMP6 regulates SMAD6 to antagonize TGFβ-SMAD3, thereby exerting an anti-fibrotic effect. Finally, we also predicted some potential herbal medicines that regulate BMP6 for the treatment of DCM, including Rhizoma Polygoni cuspidati, Acidum Citricum, and Herba Sonchi Arvensis, among others.

However, there are still some limitations in this study. Although we found that BMP6 can regulate SMAD6 to produce anti-fibrosis effects, this result may be direct or indirect regulation. Therefore, in the subsequent more in-depth research, we will use protein interaction technology to explore this regulatory relationship. In addition, due to the differences in gene expression profiles between humans and rats, and the potential errors between the DCM model constructed using DOX and real-world DCM patients, these limitations will be greatly reduced with further research.

But overall, we found that BMP6 is a potential indicator of inflammatory fibrosis in DCM hearts, whereas it functionally exerts an antifibrotic effect. Our research findings will provide novel promising pathways for the treatment of DCM.

## Conclusion

In this study, through multi-genomics analysis, mendelian randomization analysis, and in vivo and in vitro experiments on animal models, we found that BMP6 is a promising biomarker for assessing the degree of fibrosis in DCM. Furthermore, in our functional study of BMP6, we identified it as a protective factor for DCM. Overall, BMP6 serves as a novel potential intervention target for achieving anti-inflammatory fibrotic therapy in DCM, representing a promising strategy.

## Supplementary Information

Additional file 1

## Data Availability

Bulk RNA transcriptome data can be accessed from the following link: https://www.ncbi.nlm.nih.gov/geo/query/acc.cgi?acc=GSE29819. Single cell sequencing data can be accessed from the following link: https://www.ncbi.nlm.nih.gov/geo/query/acc.cgi?acc=GSE145154. GWAS data can be accessed from the following link: https://gwas.mrcieu.ac.uk/. The other raw data can be found in the supplementary material, and further inquiries can be directed to the corresponding authors. HERB database can be accessed from the following link: http://herb.ac.cn/.

## Abbreviations

DCM: Dilated cardiomyopathy
NH: Non-DCM humans
DEGS: Differentially expressed genes
WGCNA: Weighted gene co-expression network analysis
ImmDEGs: Differentially expressed genes related to immunity
SenDEGS: Differentially expressed genes related to senescence
MR: Mendelian randomization
IVW: Inverse variance weighted
DOX: Doxorubicin
NC: Normal control
EF: Ejection fraction
FS: Fractional shortening

## References

[1] Savarese G, Becher PM, Lund LH, et al. Global burden of heart failure: a comprehensive and updated review of epidemiology. Cardiovasc Res. 2023;118(17):3272–87. 10.1093/cvr/cvac013. 35150240 10.1093/cvr/cvac013

[2] Joynt Maddox KE, Elkind MSV, Aparicio HJ, et al. Forecasting the Burden of Cardiovascular Disease and Stroke in the United States Through 2050-Prevalence of Risk Factors and Disease: a Presidential Advisory From the American Heart Association. Circulation. 2024;150(4):e65–88. 10.1161/cir.0000000000001256. 38832505 10.1161/CIR.0000000000001256

[3] Emmons-Bell S, Johnson C, Roth G. Prevalence, incidence and survival of heart failure: a systematic review. Heart. 2022;108(17):1351–60. 10.1136/heartjnl-2021-320131. 35042750 10.1136/heartjnl-2021-320131 PMC9380485

[4] Ciarambino T, Menna G, Sansone G, et al. Cardiomyopathies: an overview. Int J Mol Sci. 2021. 10.3390/ijms22147722. 34299342 10.3390/ijms22147722 PMC8303989

[5] Orphanou N, Papatheodorou E, Anastasakis A. Dilated cardiomyopathy in the era of precision medicine: latest concepts and developments. Heart Fail Rev. 2022;27(4):1173–91. 10.1007/s10741-021-10139-0. 34263412 10.1007/s10741-021-10139-0 PMC8279384

[6] Reichart D, Magnussen C, Zeller T, et al. Dilated cardiomyopathy: from epidemiologic to genetic phenotypes: a translational review of current literature. J Intern Med. 2019;286(4):362–72. 10.1111/joim.12944. 31132311 10.1111/joim.12944

[7] Myers MC, Breznen B, Zhong Y, et al. Diverse concepts in definitions of dilated cardiomyopathy: theory and practice. Cardiol Res. 2024;15(5):319–29. 10.14740/cr1679. 39420975 10.14740/cr1679 PMC11483116

[8] Peters S, Kumar S, Elliott P, et al. Arrhythmic genotypes in familial dilated cardiomyopathy: implications for genetic testing and clinical management. Heart Lung Circ. 2019;28(1):31–8. 10.1016/j.hlc.2018.09.010. 30482687 10.1016/j.hlc.2018.09.010

[9] Schultheiss HP, Fairweather D, Caforio ALP, et al. Dilated cardiomyopathy. Nat Rev Dis Primers. 2019;5(1):32. 10.1038/s41572-019-0084-1. 31073128 10.1038/s41572-019-0084-1 PMC7096917

[10] Seferović PM, Polovina M, Bauersachs J, et al. Heart failure in cardiomyopathies: a position paper from the Heart Failure Association of the European Society of Cardiology. Eur J Heart Fail. 2019;21(5):553–76. 10.1002/ejhf.1461. 30989768 10.1002/ejhf.1461

[11] Eldemire R, Mestroni L, Taylor MRG. Genetics of dilated cardiomyopathy. Annu Rev Med. 2024;75:417–26. 10.1146/annurev-med-052422-020535. 37788487 10.1146/annurev-med-052422-020535 PMC10842880

[12] Harding D, Chong MHA, Lahoti N, et al. Dilated cardiomyopathy and chronic cardiac inflammation: pathogenesis, diagnosis and therapy. J Intern Med. 2023;293(1):23–47. 10.1111/joim.13556. 36030368 10.1111/joim.13556

[13] Newman NA, Burke MA. Dilated cardiomyopathy: a genetic journey from past to future. Int J Mol Sci. 2024. 10.3390/ijms252111460. 39519012 10.3390/ijms252111460 PMC11546582

[14] Papamichail A, Kourek C, Briasoulis A, et al. Targeting key inflammatory mechanisms underlying heart failure: a comprehensive review. Int J Mol Sci. 2023. 10.3390/ijms25010510. 38203681 10.3390/ijms25010510 PMC10778956

[15] Reina-Couto M, Pereira-Terra P, Quelhas-Santos J, et al. Inflammation in human heart failure: major mediators and therapeutic targets. Front Physiol. 2021;12: 746494. 10.3389/fphys.2021.746494. 34707513 10.3389/fphys.2021.746494 PMC8543018

[16] Uccello G, Bonacchi G, Rossi VA, et al. Myocarditis and chronic inflammatory cardiomyopathy, from acute inflammation to chronic inflammatory damage: an update on pathophysiology and diagnosis. J Clin Med. 2023. 10.3390/jcm13010150. 38202158 10.3390/jcm13010150 PMC10780032

[17] Cojan-Minzat BO, Zlibut A, Agoston-Coldea L. Non-ischemic dilated cardiomyopathy and cardiac fibrosis. Heart Fail Rev. 2021;26(5):1081–101. 10.1007/s10741-020-09940-0. 32170530 10.1007/s10741-020-09940-0

[18] Legere SA, Haidl ID, Légaré JF, et al. Mast cells in cardiac fibrosis: new insights suggest opportunities for intervention. Front Immunol. 2019;10:580. 10.3389/fimmu.2019.00580. 31001246 10.3389/fimmu.2019.00580 PMC6455071

[19] Zhang J, Ji C, Zhai X, et al. Frontiers and hotspots evolution in anti-inflammatory studies for coronary heart disease: a bibliometric analysis of 1990–2022. Front Cardiovasc Med. 2023;10:1038738. 10.3389/fcvm.2023.1038738. 36873405 10.3389/fcvm.2023.1038738 PMC9978200

[20] Gaertner A, Schwientek P, Ellinghaus P, et al. Myocardial transcriptome analysis of human arrhythmogenic right ventricular cardiomyopathy. Physiol Genomics. 2012;44(1):99–109. 10.1152/physiolgenomics.00094.2011. 22085907 10.1152/physiolgenomics.00094.2011

[21] Newman AM, Steen CB, Liu CL, et al. Determining cell type abundance and expression from bulk tissues with digital cytometry. Nat Biotechnol. 2019;37(7):773–82. 10.1038/s41587-019-0114-2. 31061481 10.1038/s41587-019-0114-2 PMC6610714

[22] Saul D, Kosinsky RL, Atkinson EJ, et al. A new gene set identifies senescent cells and predicts senescence-associated pathways across tissues. Nat Commun. 2022;13(1):4827. 10.1038/s41467-022-32552-1. 35974106 10.1038/s41467-022-32552-1 PMC9381717

[23] Rao M, Wang X, Guo G, et al. Resolving the intertwining of inflammation and fibrosis in human heart failure at single-cell level. Basic Res Cardiol. 2021;116(1):55. 10.1007/s00395-021-00897-1. 34601654 10.1007/s00395-021-00897-1

[24] Shen LJ, Lu S, Zhou YH, et al. Developing a rat model of dilated cardiomyopathy with improved survival. J Zhejiang Univ Sci B. 2016;17(12):975–83. 10.1631/jzus.B1600257. 27921402 10.1631/jzus.B1600257 PMC5172601

[25] Caviedes Bottner P, Córdova Fernández T, Larraín Valenzuela M, et al. Dilated cardiomyopathy and severe heart failure. An update for pediatricians. Arch Argent Pediatr. 2018;116(3):e421–8. 10.5546/aap.2018.eng.e421. 29756716 10.5546/aap.2018.eng.e421

[26] Lian H, Song S, Chen W, et al. Genetic characterization of dilated cardiomyopathy patients undergoing heart transplantation in the Chinese population by whole-exome sequencing. J Transl Med. 2023;21(1):476. 10.1186/s12967-023-04282-5. 37461109 10.1186/s12967-023-04282-5 PMC10351148

[27] Li H, Hastings MH, Rhee J, et al. Targeting age-related pathways in heart failure. Circ Res. 2020;126(4):533–51. 10.1161/circresaha.119.315889. 32078451 10.1161/CIRCRESAHA.119.315889 PMC7041880

[28] Cesselli D, Aleksova A, Mazzega E, et al. Cardiac stem cell aging and heart failure. Pharmacol Res. 2018;127:26–32. 10.1016/j.phrs.2017.01.013. 28111264 10.1016/j.phrs.2017.01.013

[29] Osorio JM, Espinoza-Pérez C, Rimassa-Taré C, et al. Senescent cardiac fibroblasts: a key role in cardiac fibrosis. Biochim Biophys Acta Mol Basis Dis. 2023;1869(4): 166642. 10.1016/j.bbadis.2023.166642. 36669578 10.1016/j.bbadis.2023.166642

[30] Hanna A, Frangogiannis NG. Inflammatory cytokines and chemokines as therapeutic targets in heart failure. Cardiovasc Drugs Ther. 2020;34(6):849–63. 10.1007/s10557-020-07071-0. 32902739 10.1007/s10557-020-07071-0 PMC7479403

[31] Paulus WJ, Zile MR. From systemic inflammation to myocardial fibrosis: the heart failure with preserved ejection fraction paradigm revisited. Circ Res. 2021;128(10):1451–67. 10.1161/circresaha.121.318159. 33983831 10.1161/CIRCRESAHA.121.318159 PMC8351796

[32] Antar SA, Ashour NA, Marawan ME, et al. Fibrosis: types, effects, markers, mechanisms for disease progression, and its relation with oxidative stress, immunity, and inflammation. Int J Mol Sci. 2023. 10.3390/ijms24044004. 36835428 10.3390/ijms24044004 PMC9963026

[33] Xue T, Qiu X, Liu H, et al. Epigenetic regulation in fibrosis progress. Pharmacol Res. 2021;173: 105910. 10.1016/j.phrs.2021.105910. 34562602 10.1016/j.phrs.2021.105910

[34] Liu Y, Xu X, Lei W, et al. The NLRP3 inflammasome in fibrosis and aging: the known unknowns. Ageing Res Rev. 2022;79: 101638. 10.1016/j.arr.2022.101638. 35525426 10.1016/j.arr.2022.101638

[35] Hara A, Tallquist MD. Fibroblast and immune cell cross-talk in cardiac fibrosis. Curr Cardiol Rep. 2023;25(6):485–93. 10.1007/s11886-023-01877-8. 37074566 10.1007/s11886-023-01877-8

[36] Lu G, Ge Z, Chen X, et al. BMP6 knockdown enhances cardiac fibrosis in a mouse myocardial infarction model by upregulating AP-1/CEMIP expression. Clin Transl Med. 2023;13(6): e1296. 10.1002/ctm2.1296. 37313693 10.1002/ctm2.1296 PMC10265437

[37] Hata A, Chen YG. TGF-β signaling from receptors to Smads. Cold Spring Harb Perspect Biol. 2016. 10.1101/cshperspect.a022061. 27449815 10.1101/cshperspect.a022061 PMC5008074

[38] Luo K. Signaling cross talk between TGF-β/Smad and other signaling pathways. Cold Spring Harb Perspect Biol. 2017. 10.1101/cshperspect.a022137. 27836834 10.1101/cshperspect.a022137 PMC5204325

[39] Chia ZJ, Kumarapperuma H, Zhang R, et al. Smad transcription factors as mediators of 7 transmembrane G protein-coupled receptor signalling. Acta Pharmacol Sin. 2024. 10.1038/s41401-024-01413-6. 39506064 10.1038/s41401-024-01413-6 PMC11950520

[40] Tzavlaki K, Moustakas A. TGF-β signaling. Biomolecules. 2020. 10.3390/biom10030487. 32210029 10.3390/biom10030487 PMC7175140

[41] Miyazawa K, Miyazono K. Regulation of TGF-β family signaling by inhibitory smads. Cold Spring Harb Perspect Biol. 2017. 10.1101/cshperspect.a022095. 27920040 10.1101/cshperspect.a022095 PMC5334261

[42] Hu HH, Chen DQ, Wang YN, et al. New insights into TGF-β/Smad signaling in tissue fibrosis. Chem Biol Interact. 2018;292:76–83. 10.1016/j.cbi.2018.07.008. 30017632 10.1016/j.cbi.2018.07.008

[43] Wu M, Wu S, Chen W, et al. The roles and regulatory mechanisms of TGF-β and BMP signaling in bone and cartilage development, homeostasis and disease. Cell Res. 2024;34(2):101–23. 10.1038/s41422-023-00918-9. 38267638 10.1038/s41422-023-00918-9 PMC10837209

[44] Nickel J, Mueller TD. Specification of BMP signaling. Cells. 2019. 10.3390/cells8121579. 31817503 10.3390/cells8121579 PMC6953019

[45] Khalil H, Kanisicak O, Prasad V, et al. Fibroblast-specific TGF-β-Smad2/3 signaling underlies cardiac fibrosis. J Clin Invest. 2017;127(10):3770–83. 10.1172/jci94753. 28891814 10.1172/JCI94753 PMC5617658

